# ^18^F-labeled FGFR1 peptide: a new PET probe for subtype FGFR1 receptor imaging

**DOI:** 10.3389/fonc.2023.1047080

**Published:** 2023-04-27

**Authors:** Yang Chen, Jingya Han, Yan Zhao, Xinming Zhao, Mengmeng Zhao, Jingmian Zhang, Jianfang Wang

**Affiliations:** ^1^ Department of Nuclear Medicine, The Fourth Hospital of Hebei Medical University, Shijiazhuang, China; ^2^ Department of Oncology, Shandong Provincial Hospital Affiliated to Shandong First Medical University, Jinan, China; ^3^ Department of Oncology, The Fourth Hospital of Hebei Medical University, Shijiazhuang, China; ^4^ Hebei Provincial Key Laboratory of Tumor Microenvironment and Drug Resistance, Shijiazhuang, China

**Keywords:** targeting peptide, fluorine-18, molecular imaging, fibroblast growth factor receptor (FGFR), malignant tumor

## Abstract

**Introduction:**

The fibroblast growth factor receptor (FGFR) family is highly expressed in a variety of tumor types and represents a new target for cancer therapy. Different FGFR subtype aberrations have been found to exhibit highly variable sensitivity and efficacy to FGFR inhibitors.

**Methods:**

The present study is the first to suggest an imaging method for assessing FGFR1 expression. The FGFR1-targeting peptide NOTA-PEG2-KAEWKSLGEEAWHSK was synthesized by manual solid-phase peptide synthesis and high-pressure liquid chromatography (HPLC) purification and then labeled with fluorine-18 using NOTA as a chelator. *In vitro* and *in vivo* experiments were conducted to evaluate the stability, affinity and specificity of the probe. Tumor targeting efficacy and biodistribution were evaluated by micro-PET/CT imaging in RT-112, A549, SNU-16 and Calu-3 xenografts.

**Results:**

The radiochemical purity of [18F]F-FGFR1 was 98.66% ± 0.30% (n = 3) with excellent stability. The cellular uptake rate of [18F]F-FGFR1 in the RT-112 cell line (FGFR1 overexpression) was higher than that in the other cell lines and could be blocked by the presence of excess unlabeled FGFR1 peptide. Micro-PET/CT imaging revealed a significant concentration of [18F]F-FGFR1 in RT-112 xenografts with no or very low uptake in nontargeted organs and tissues, which demonstrated that [18F]F-FGFR1 was selectively taken up by FGFR1-positive tumors.

**Conclusion:**

[18F]F-FGFR1 showed high stability, affinity, specificity and good imaging capacity for FGFR1-overexpressing tumors *in vivo*, which provides new application potential in the visualization of FGFR1 expression in solid tumors.

## Introduction

Malignant tumors are a major public health problem and pose a serious threat to human health worldwide (1). Patient stratification and individualized precision therapy strategies rely on the identification of molecular alterations that may be causative agents in tumorigenesis and tumor growth. Fibroblast growth factor receptor (FGFR) signaling is currently an evolving research field and relative researches have revealed it’s higher heterogeneity than classical BRAF, ALK or EGFR signaling (2).

The FGFR family consists of 4 highly conserved transmembrane tyrosine kinase receptors (RTKs), which interact with 18 soluble fibroblast growth factors (FGFs) (3–6). The deregulation of FGF/FGFR signaling has been implicated in tumorigenesis, invasion, angiogenesis, metastasis, recurrence, epithelial-mesenchymal transition (EMT) and resistance to anticancer therapy (7–16). In multiple cancer types, including lung, bladder, breast, glioblastoma, ovarian and prostate cancer, the FGF/FGFR signaling axis in oncogenesis is mainly mediated by oncogenic FGFR-encoding gene alterations (receptor amplification, translocation or rearrangements), with the majority being FGFR1 amplification (17).

Since FGF/FGFR signaling is important in cancer, abundant efforts have been dedicated to its efficient inhibition, leading to an almost simultaneous release of competing targeting peptides and drug candidates (2, 18–20). In addition to monoclonal antibodies (mAbs) against FGFR and FGF ligand traps, the first generation of FGFR inhibitors, multikinase inhibitors, not only combine with FGFR but also target several growth factor receptors, such as DGFRα/β, RET and VEGFR1/3 (19). Second-generation FGFR inhibitors are reported to have a higher selectivity and a lower level of toxicological side effects (3). Currently, FGFR-targeted therapies have not been approved for clinical application yet, although some clinical trials have reported significant survival benefits, some others have drawn the opposite conclusions (21). Now evidences have unveiled that different FGFR subtype aberrations exhibited highly varied sensitivity and efficacy to FGFR inhibitors (22). However, the FGFR subtypes have not been identified and distinguished in patients who were enrolled in the early-phase clinical trials. Besides, the aberrant FGFR targeting process is complex and dynamic. Thus, in clinical trials, one of the major challenges is to prospectively select patients with specific FGFR aberrations.

To assess FGFR aberration status, tissue specimens generally need to be collected *via* invasive procedures, such as surgery or biopsy. These approaches may be limited by heterogeneity, tumor stage, the invasiveness of the procedure, long operating time and the functional status of patients’ major organs. To overcome the inconvenience of invasive operations, molecular imaging has emerged as a noninvasive method to detect FGFR expression in tumors at the cellular or subcellular level (23–25), which are different from traditional anatomic imaging. These methods are different from traditional anatomic imaging. Because many selective FGFR inhibitors, such as AZD4547, NVP-BJG398 and JNJ-42756493, have shown higher affinity for FGFR1 (IC_50_ < 1 nM) than for other subtypes of FGFR in preclinical studies (7), FGFR1-targeted imaging is emerging as a potentially promising approach for detecting and treating FGFR1-positive tumors.

Monoclonal antibodies, antibody fragments and peptides have frequently been used as ligands for positron labeling. Among the different strategies, the most viable approach has been the utilization of peptide-based probes. The lower molecular weight and the faster clearance of plasma have produced favorable target-to-nontarget ratios and excellent imaging effects (26). However, key molecular probes that target FGFR1 for this approach remain unidentified.

Neural cell adhesion molecule (NCAM) was proven to influence cell migration, proliferation, and differentiation, which may due to the functions of the adhesive property as well as the signaling property (27). The two membrane-proximal F3 modules of NCAM encompassing the F and G b-strands and the interconnecting loop region have been shown to be involved in FGFR binding. A publicly available patent (European Patent EP1765861) compounded new peptides derived from the sequence of the proximal F3 modules of NCAM that are capable of binding to FGFR. Among them, KAEWKSLGEEAWHSK was the preferred sequence with the best character and binding capacity to FGFR, especially to FGFR1. However, whether the sequence was feasible for nuclear imaging remained unclear.

Based on the clinical demand and current understanding of FGFR mentioned above, this project amied to develop a positron-emitting isotope ^18^F-labeled FGFR1 targeting peptide ([^18^F]F-FGFR1) with advantages in rapid uptake and highly specific affinity, and visualize FGFR1 expression by clear imaging in xenografts, which may lay the foundation for distinguishing FGFR1-positive-patients in a noninvasive and reproducible way.

## Material and methods

### Synthesis and radiolabeling

The FGFR1-targeting peptide KAEWKSLGEEAWHSK was one of the sequences with the best characteristics obtained from a publicly available patent (European Patent EP1765861). In this study, NOTA-PEG2-KAEWKSLGEEAWHSK was synthesized by solid-phase synthesis and purified through high-pressure liquid chromatography (HPLC) (Shanghai Science Peptide Biological Technology Co., LTD, China). The HPLC column was an Agela (250*4.6 mm I.D.) C18 column. The detection wavelength was 220 nm. Then, 0.05% trifluoroacetic acid (TFA) + 2% acetonitrile was used as buffer A. Buffer B was 0.05% TFA + 90% acetonitrile. The gradient was set between 12% and 35% buffer B in 23 min. Mass spectrometry (MS) was used to identify the product.

The FGFR1-targeting peptide was labeled with fluorine-18 *via* a manual labeling method. Fluorine-18 was obtained by eluting the QMA cartridge with 0.4 ml of saline. The reaction system included fluorine-18 (100 µl, 259-370 MBq), AlCl_3_·6H_2_O (6 µl, 2 nM) and potassium hydrogen phthalate (KHP) (11 µl, 0.5 M), which were mixed thoroughly. After incubation at room temperature for 5 min, 24 µl of NOTA-PEG2-FGFR1-peptide (0.9 mM, 21.82 nmol) was added. Then, the sample was heated at a constant temperature of 110°C for 15 min. After cooling to room temperature naturally, the mixture was transferred over a Sep-Pak C18-Light cartridge (Waters. USA), which was previously activated with 10 ml of anhydrous ethanol followed by 10 ml of distilled water. The fluorine-18 was eluted from the Sep-Pak C18-Light cartridge with 15 ml of distilled water. Ultimately, the product was obtained by solid-phase extraction with 0.5 ml of 80% ethanol. The ^18^F-labeled FGFR1-targeting peptide tracer was assessed with gradient reversed-phase high-pressure liquid chromatography (RP-HPLC), which used the mobile phases (A) 20-80% acetonitrile containing 0.1% TFA in 20 min and (B) 0.1% TFA-H_2_O.

### Cell culture

The human lung adenocarcinoma cell line Calu-3 (FGFR1-low expression) was kindly provided by the Stem Cell Bank, Chinese Academy of Sciences (China) and maintained in MEM (BI, Israel) containing 10% fetal bovine serum (FBS) (BI, Israel). RPMI 1640 medium (BI, Israel) supplemented with 10% FBS was used to culture the gastric carcinoma cell line SNU-16 (FGFR1-low expression) purchased from KeyGEN BioTECH (China), the lung adenocarcinoma cell line A549 (FGFR1-low expression) and the bladder cancer cell line RT-112 (FGFR1-high expression), purchased from Procell (China). All cell lines were identified by short tandem repeat (STR) profiling before purchase and Mycoplasma testing was negative. The cells were then cultured under standard conditions (37°C in a 5% CO_2_ atmosphere). The growth rate and morphology of all cell lines were determined by inverted microscopy with phase contrast. After trypsin-EDTA solution (0.02% EDTA, 0.25% trypsin; BI, Israel) digestion of the adherent cells, the cells were collected. All experiments were performed at the first five passages of the cells.

### Western blot analysis

Total proteins were extracted from cells using cell lysis buffer. A BCA Protein Assay Kit (Solarbio, China) was used to quantify the total protein. The PVDF membranes (Millipore, USA) were presoaked in methanol for 5 min. Protein was separated by SDS−PAGE and transferred to PVDF membranes. After transformation, membranes were blocked for 2 h at room temperature with 5% BSA (Solarbio, China) in Tris-buffered saline containing Tween-20 (TBST, pH 7.6). FGFR1-4 primary antibodies were purchased from Cell Signaling Technology, Inc. (CST, USA) with catalog numbers 9740S, 23328S, 4574S and 8562S. The FGFR1-4 primary antibodies were diluted to 1:1000, and GAPDH was diluted to 1:10000 with blocking solution (5% BSA) and subsequently incubated on a shaker at 4°C overnight. The membranes were incubated for 1 h at room temperature with HRP-labeled anti-rabbit secondary antibody (Abcam, UK) diluted 1:5000, and washed with TBST three times; signals were then detected by chemiluminescence. Signals from immunoblots were obtained from three independent experiments and quantified with ImageJ (NIH). The relative expression of FGFR1 was evaluated based on the gray value of the FGFR1 protein/the gray value of GAPDH.

### *In vitro* and *in vivo* stability analysis

The *in vitro* and *in vivo* stability of [^18^F]F-FGFR1 was evaluated by RP-HPLC analysis. In the *in vitro* stability test, the labeled tracer samples were mixed with sterile saline and fresh human serum and then incubated at 37°C for 1, 2 and 4 h. The radiochemical purity of the final compounds was determined by RP-HPLC.

For the *in vivo* radiochemical stability experiment, urine samples were obtained from xenograft nude mice 60 min after injection of 150 µl (7.4 MBq, 2.53 GBq/μmol) [^18^F]F-FGFR1 and analyzed by RP-HPLC.

### Cellular uptake, internalization and blocking studies

RT-112, A549, SNU-16 and Calu-3 cells were cultured at a density of 1*10^5^ cells per well in 24-well plates the day before cellular uptake experiments. Cells were rinsed twice with 1000 µl of phosphate-buffered saline (PBS) (BI, Israel), followed by 1000 µl of growth medium. Then, 30 µl (111 KBq, 1.81-2.53 GBq/μmol) of [^18^F]F-FGFR1 was added to each well, and the cells were incubated with the peptide for 15, 30, 60 and 120 min. At each cutoff time, 6 wells of supernatant were collected. The collected wells were washed twice with 1000 µl of PBS. Radioactivity counts were marked as Cout. SNU-16, the cells in suspension did not need to be digested with trypsin-EDTA and were centrifuged at 1000 g for 2 min in each step. After 500 µl of trypsin-EDTA digestion of the adherent cells and washed twice with 1000 µl of PBS, the collected cells were defined as Cin. Radioactivity was counted with an automatic γ counter. The formula for the cell uptake rate was as follows: Cin/(Cin + Cout).

Internalization studies were performed according to the planned experiments. Thirty microliters (111 KBq, 1.81-2.53 GBq/μmol) of [^18^F]F-FGFR1 was incubated with fresh medium in 24-well plates. The medium was discarded at predetermined time points, and the wells were rinsed twice with 1000 µl of PBS. Then, 1000 µl of acetate buffer (0.2 M acetic acid/0.5 M NaCl, pH 2.5) was added to the wells to remove surface-bound radioactivity. After removing the acetate buffer, the cells were washed twice with 1000 µl of PBS. The acetate buffer and PBS were collected and marked as Cs. The radioactivity of the collected cell lysate was measured and defined as Cin. The internalization rate was assessed in a similar method to the cell uptake rate calculation: Cin/(Cin + Cs).

To determine the specific binding of [^18^F]F-FGFR1 with FGFR1 on the cell surface, 200-fold unlabeled peptide (220 µg) and 30 µl of [^18^F]F-FGFR1 (111 KBq) were added to RT-112 cells at the same time. The supernatants were removed after 120 min of incubation. The subsequent steps of cell collection and radioactivity detection were performed as described above. The cell uptake rate was calculated by Cin/(Cin + Cout).

### Saturation binding assay

RT-112 cells were cultured at a density of 6*10^5^ cells per well in 48-well plates the day before cellular uptake experiments. Different concentration gradients of [^18^F]F-FGFR1 (0.005 µM-0.5 µM) were added to one 48-well plate, and 200-fold unlabeled peptide was added to another. After 60 min of incubation, the radioactivity of cells in each well was recorded by an automatic γ counter, and the equilibrium dissociation constants (*K*_D_) were measured by GraphPad Prism v5.0 (GraphPad Software Inc., USA).

### Animal xenograft model

All animal experiments were carried out in accordance with the Laboratory Animal Ethical Review of Animal Guidelines of China. BALB/c nude mice (sex, female; age, 4 weeks, body weight range, 19-21 g; China) were used for this study. Four cell lines (2*10^7^ cells/nude mouse) were subcutaneously injected into the right forelimb to induce solid tumors. All xenograft nude mice were housed in laminar flow cabinets in a specific pathogen-free (SPF) environment and were provided ad libitum access to food and water at the Laboratory Animal Center of the Fourth Hospital of Hebei Medical University (China). The indication for micro-PET/CT imaging was xenografts with a tumor volume of 50-130 mm^3^. All animal experiments strictly adhered to the Principles of the Laboratory Animal Ethical Committee of the Fourth Hospital Hebei Medical University (No. 2020016).

### Molecular imaging and biodistribution

Xenograft mice (n=5) were fastly injected of 200 µl of [^18^F]F-FGFR1 (11.1 MBq) through tail vein. For the *in vivo* blocking study, RT-112 (FGFR1-high expression) tumor-bearing mice (n = 5) were injected with a tracer that was mixed with 200-fold (1.58 mg) unlabeled peptide to saturate the receptors in the tumors. Mice were anesthetized *via* 3% isoflurane inhalation (RWD Life Science Inc. China) for induction and 2% isoflurane inhalation for maintenance. Micro-PET/CT imaging was performed on a micro-PET/SPECT/CT machine (NOVEL MEDICAL Equipment Ltd. China). Whole-body scans were performed 30, 60 and 120 min after tracer administration. The CT scanning parameters were 80 kV, 0.5 mA, 2000 ms/frames, and 360 frames; the images were reconstructed with a 512*512 matrix and a slice thickness of 0.18 mm. PET images were acquired with a 140*140 matrix and 10 min of total acquisition time. Reconstructed images were processed with the comprehensive image analysis software Pmod v4.201 (PMOD Technologies LLC. Switzerland). The biodistribution of the major organs (brain, heart, lung, liver, kidney, bone and muscle) and tumor were analyzed by manually outlining the regions of interest (ROIs) of the entire organs and tissue at each time point of the micro-PET/CT scan. The radioactivity accumulated in bone and muscle was measured in the limbs without xenograft tumors.

### Statistical analysis

Numerical variables are expressed as the mean ± standard deviation (**x̄** ± SD) and were compared using Student’s t test between two different groups. Multiple groups were compared by one-way ANOVA. If the data did not conform to a normal distribution and if the variance criteria were not homogeneous, nonparametric tests with rank transformation were performed. *P* values of less than 0.05 (*P* < 0.05) were considered statistically significant.

## Results

### Synthesis and radiolabeling

**Figure 1:**
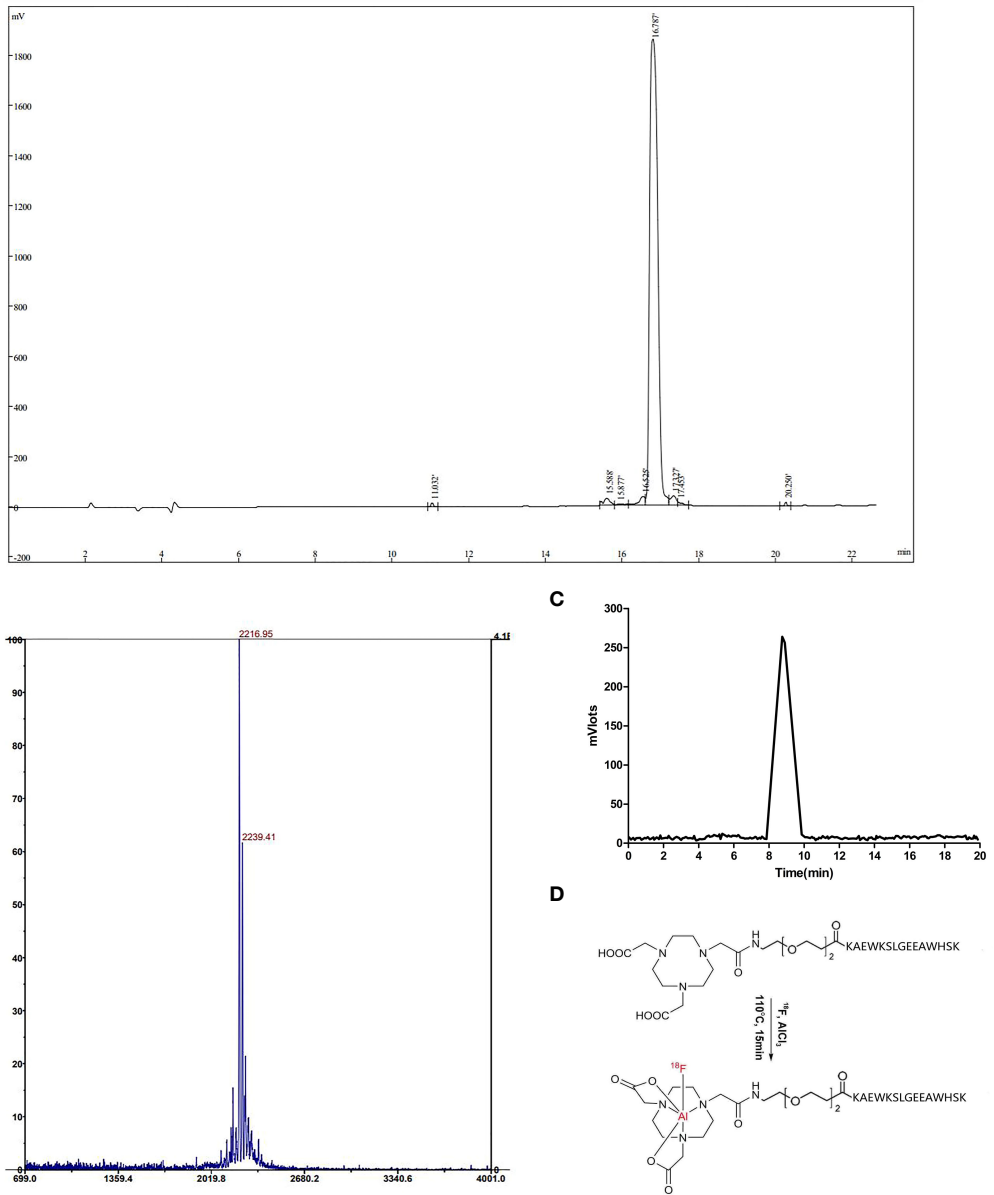
**(A)** The UV absorbance chromatogram detected by HPLC analysis showed that the purity of the FGFR1 peptide was more than 95%. **(B)** Molecular weights by MS, M+H^+^ and M+Na^+^ were 2216.95 and 2239.41, respectively. **(C)** The radiotracer absorbance chromatogram results of [^18^F]F-FGFR1 showed a single and sharp peak with a retention time of 8.7 min. **(D)** The chemical structure of NOTA-PEG2-FGFR1-peptide and [^18^F]F-FGFR1.

According to the sequence of NOTA-PEG2-KAEWKSLGEEAWHSK, peptides were synthesized by solid-phase synthesis. The purity of the FGFR1 peptide, as identified by HPLC, exceeded 95% (**Figure 1A**). The molecular weights measured by MS, M+H^+^ and MS, M+Na^+^ were 2216.95 and 2239.41, respectively (**Figure 1B**), almost being consistent with the theoretical molecular weight 2216.60, which further validated the successful synthesis of the peptide. The radiochemical purity was 98.66% ± 0.30% (n = 3), detected as a single and sharp peak on RP-HPLC with a retention time of approximately 8.7 min (**Figure 1C**) with the ideal chemical structure (**Figure 1D**). The maximum nondecay corrected radiochemical yield was 17.21%. The specific activity of [^18^F]F-FGFR1 was roughly calculated as 1.81-2.85 GBq/μmol (n > 10).

### Western blot analysis

**Figure 2:**
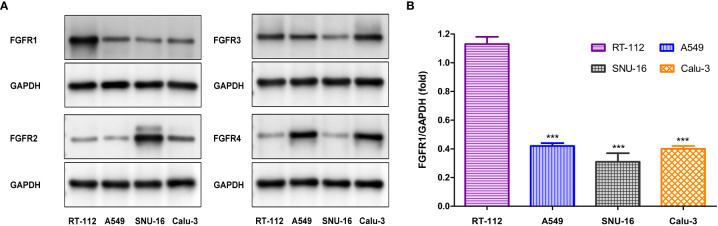
**(A)** Western blot analysis the subtype of FGFR expression in four cell lines. **(B)** FGFR1 expression (the gray value of FGFR1/GAPDH) differed significantly between RT-112 cells and other cells. ***P< 0.001.

To further validate the targeting of [^18^F]F-FGFR1 on specific subtypes of FGFR, four cell lines were screened out with different FGFR subtype expression. Western blot analysis was performed to further investigate receptor expression. The results determined that the expression levels of FGFR1-4 varied among these cell lines. FGFR1 expression was significantly higher in RT-112 cell line, compared with that in other cell lines (**Figure 2**).

### *In vitro* and *in vivo* stability analysis

**Figure 3:**
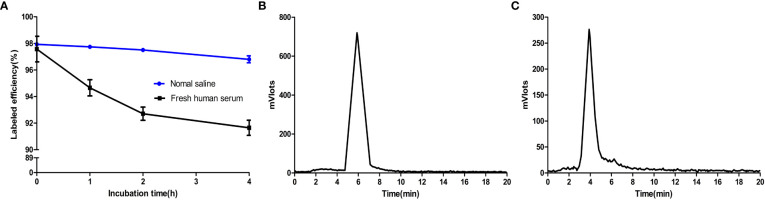
**(A)** Radiochemical stability of [^18^F]F-FGFR1 *in vitro* was greater than 90% over the period tested. **(B)** Urine samples post-injection of [^18^F]F-FGFR1 tested by RP-HPLC revealed one peak at 5.7 min. **(C)** RP-HPLC retention time of fluorine-18 was 4 min, which did not correspond to the urine samples, showed the good stability of [^18^F]F-FGFR1 *in vivo*.

Before the exploration of functional experiments *in vitro* and *in vivo*, the stability of [^18^F]F-FGFR1 was examined *in vitro* and *in vivo*. The *in vitro* stability of [^18^F]F-FGFR1 was evaluated by incubation in normal saline and fresh human serum at 37°C for 1, 2, 4 and 6 h. The radiochemical purity of the mixtures exceeded 90%, as verified by analytical RP-HPLC analysis (**Figure 3A**). Urine samples were collected from xenograft nude mice 60 min after the injection of [^18^F]F-FGFR1 for the *in vivo* radiochemical stability experiment. RP-HPLC revealed one peak, which occurred at 5.7 min (**Figure 3B**); no peak corresponded to fluorine-18 (**Figure 3C**). The results indicated that [^18^F]F-FGFR1 was stable over the period tested. Good stability laid a foundation for the cell and animal experiments.

### Cellular uptake, internalization, blocking analysis and saturation binding assay

**Figure 4:**
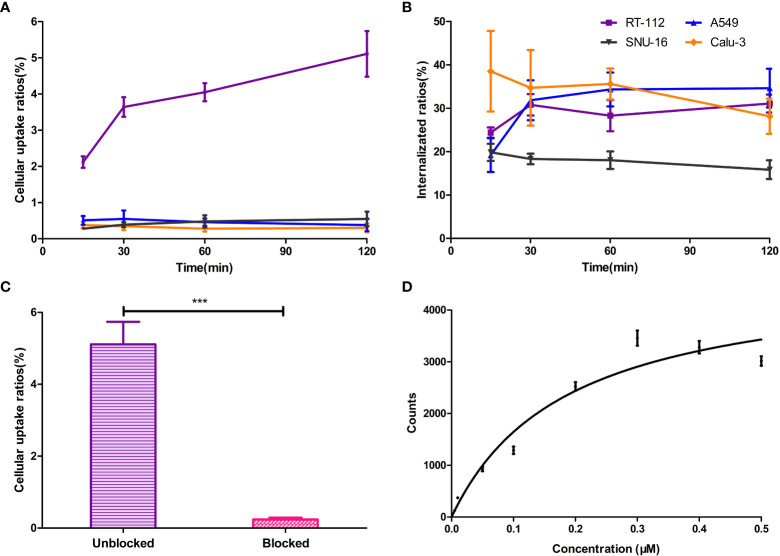
**(A)** Cellular uptake of the [^18^F]F-FGFR1 was consistently higher in FGFR1 high expressed RT-112 cell line than in others. **(B)** The internalization fraction was not significantly different in the four cell lines. **(C)** FGFR binding was blocked with excess unlabeled FGFR peptide in RT-112 cells (FGFR1-high expression). The uptake ratios decreased from 5.11 ± 0.63 to 0.24 ± 0.05. **(D)** The saturation curve of [^18^F]F-FGFR1 binding to RT-112 cells (FGFR1-high expression). The *K*_D_ was 0.19 ± 0.03 µM. (n=6, ***P < 0.001).

Live-cell experiments were carried out to assess the *in vitro* FGFR1-targeting ability of [^18^F]F-FGFR1. Cellular uptake of the labeled peptide was consistently higher in RT-112 cell line (FGFR1-high expression) than in A549, SNU-16 and Calu-3 cells (FGFR1-low expression) (*P* < 0.001, n = 6) (**Figure 4A**). The cellular internalization results are summarized in **Figure 4B**. The internalization fraction was not significantly different in the four cell lines (*P* > 0.5, n = 6). The binding of FGFR1 to radioactive probes was successfully blocked in the presence of 200-fold excess unlabeled peptide in RT-112 cells (FGFR1-high expression) (5.11 ± 0.63 *vs.* 0.24 ± 0.05, *P* < 0.001, n = 6, **Figure 4C**). The specific binding saturation curve of [^18^F]F-FGFR1 followed a concentration-dependent manner. [^18^F]F-FGFR1 exhibited high affinity and specific binding capacity to the FGFR1 on surface of RT-112 cells, *K*_D_ = 0.19 ± 0.03 µM (**Figure 4D**). The results of the experiments showed that [^18^F]F-FGFR1 could specifically target FGFR1 with a high affinity. However, it could not be internalized by FGFR1 high expression cells.

### Micro-PET/CT imaging and biodistribution analysis

**Figure 5:**
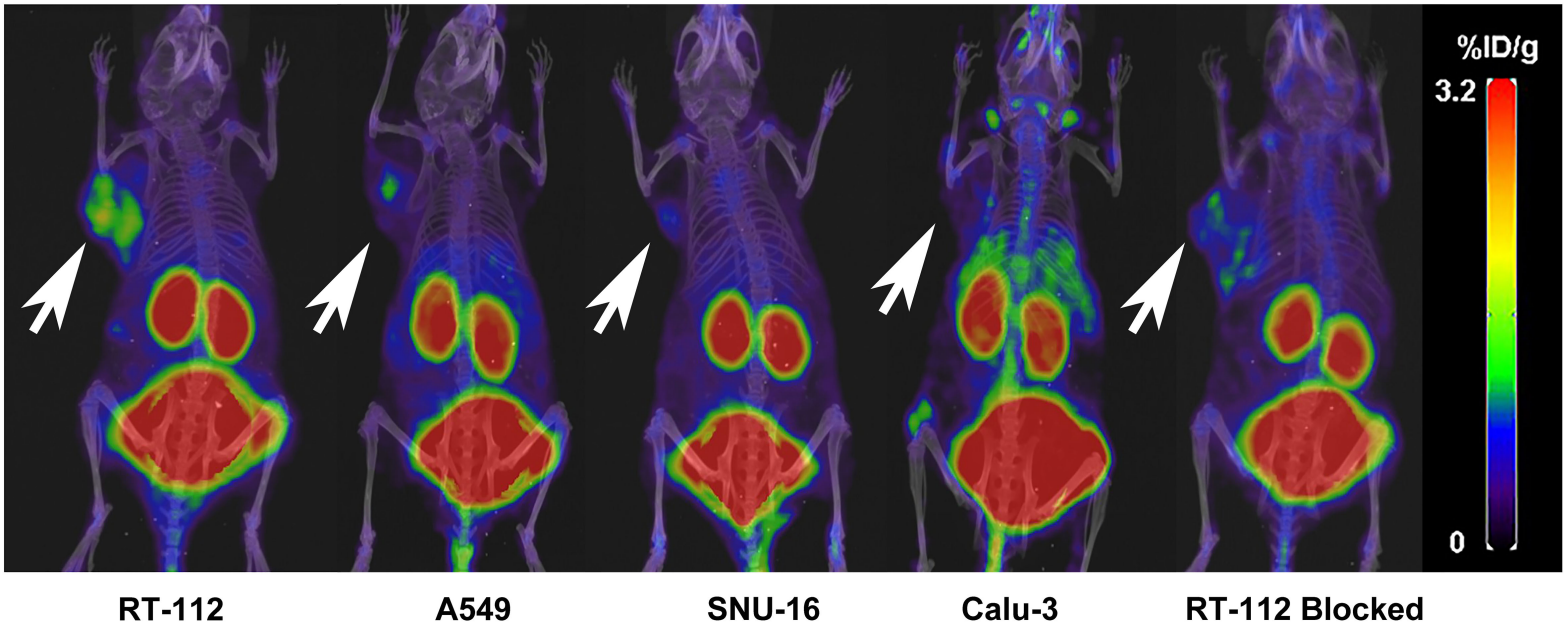
Comparing RT-112 (FGFR1-high expression) xenografts, the other xenografts showed lower tumor uptake 30 min post-injection (3D MIP of micro-PET/CT imaging). Arrows indicate tumors.

**Figure 6:**
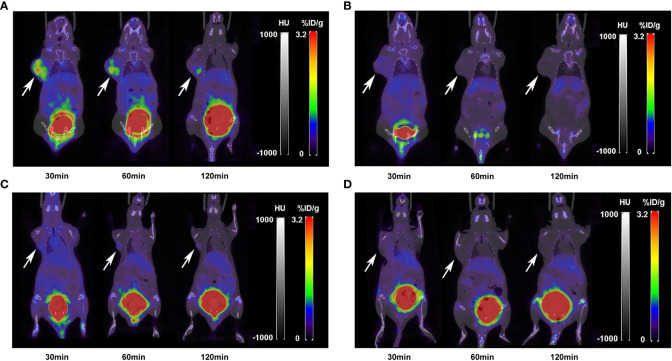
Micro-PET/CT imaging of **(A)** RT-112, **(B)** A549, **(C)** SNU-16 and **(D)** Calu-3 xenografts injected with 11.1 MBq [^18^F]F-FGFR1 at different time points. [^18^F]F-FGFR1 revealed tracer uptake in the kidney, bladder and RT-112 (FGFR1-high expression) cell-derived tumors. Arrows indicate tumors.

**Figure 7:**
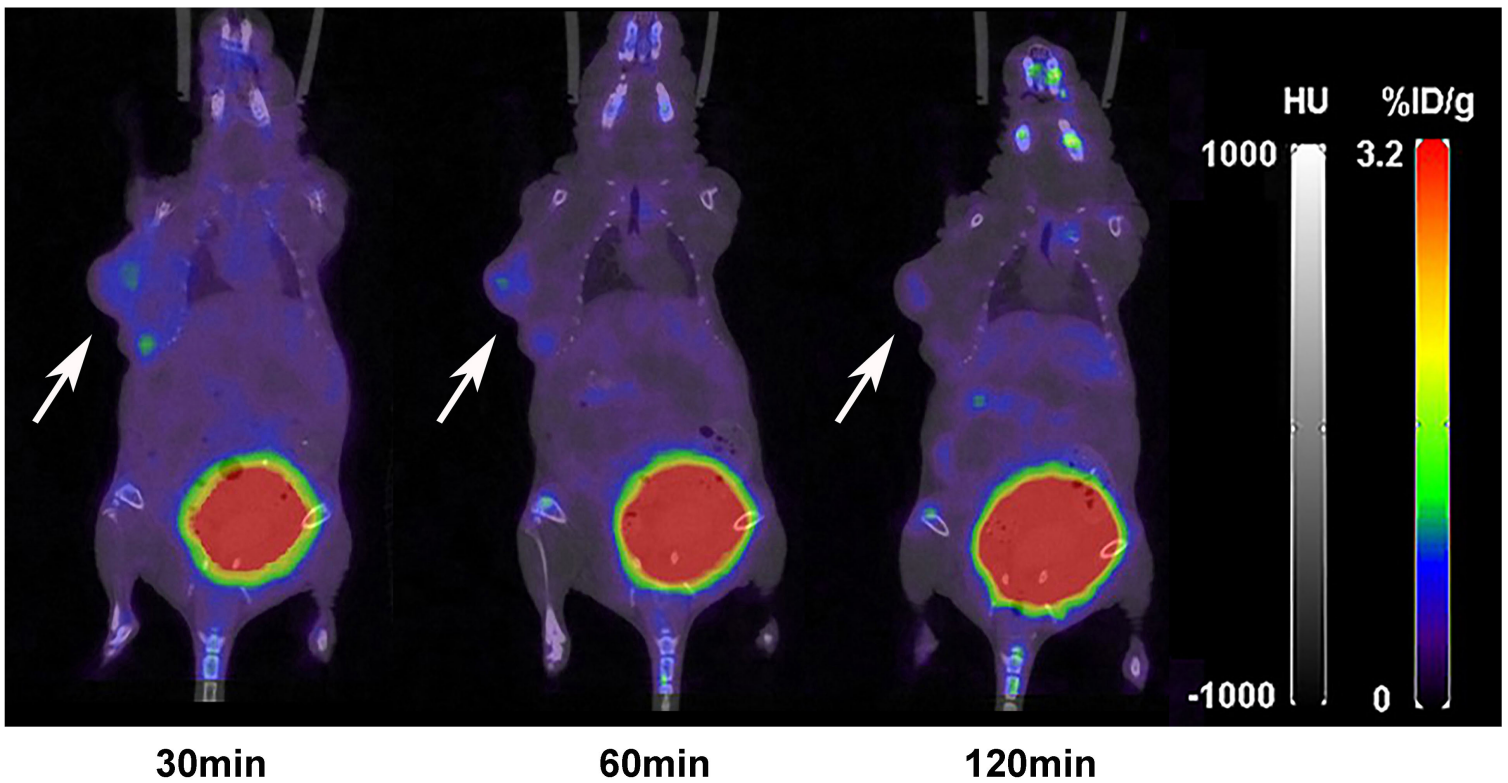
Micro-PET/CT imaging of RT-112 (FGFR1-high expression)-blocked xenografts injected with 11.1 MBq [^18^F]F-FGFR1 at different time points. The accumulation of [^18^F]F-FGFR1 in tumors were markedly decreased. Arrows indicate tumors.

The nuclear imaging of xenografts intuitively evaluated the *in vivo* distribution, metabolism and excretion properties. As shown by the 3D MIP of micro-PET/CT imaging in **Figure 5**, FGFR1-high expression RT-112 xenograft mice had significantly higher tumor uptake (3.84 ± 0.17%ID/g) than that of FGFR1-low expression A549 (0.87 ± 0.08%ID/g), SNU-16 (0.66 ± 0.05%ID/g), Calu-3 (0.59 ± 0.08%ID/g) and RT-112 blocked xenografts (0.40 ± 0.02%ID/g), at 30 min post injection. After the administration of [^18^F]F-FGFR1 in RT-112 tumor-bearing mice (**Figure 6A**), micro-PET/CT imaging revealed tracer uptake in the tumor, kidney and bladder at 30 min, 60 min and 120 min post-injection. As shown in **Figures 6B–D**, A549, SNU-16 and Calu-3 xenograft mice had lower tumor uptake at each time point after injection. Significant reductions in tumor accumulation were detected after 200-fold unlabeled peptide blockade (**Figure 7**).

**Figure 8:**
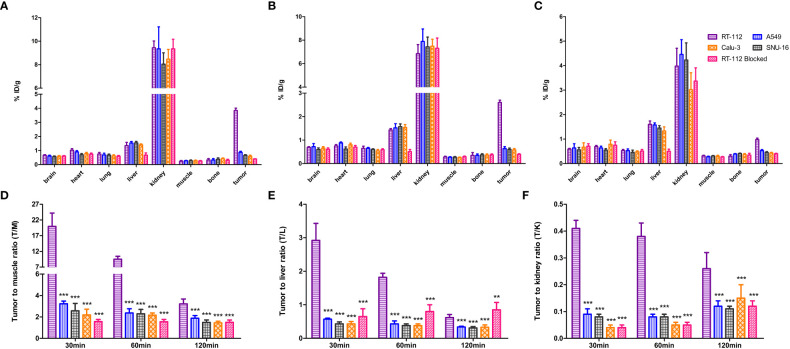
Biodistribution analysis at **(A)** 30 min, **(B)** 60 min and **(C)** 120 min post-injection of [^18^F]F-FGFR1. Quantified results of T/M **(D)**, T/L **(E)** and T/K **(F)** of the xenografts. Tumor uptake of [^18^F]F-FGFR1 in RT-112 (FGFR1-high expression) tumor-bearing mice was significantly higher than that in the others (FGFR1-low expression). (n=5, compared to RT-112 xenografts, ***P<0.001, **P<0.01).

The biodistribution data as presented in **Figure 8** and **Tables 1**–**5**, indicated that [^18^F]F-FGFR1 mainly accumulated in the kidney and RT-112, FGFR1-high expression tumors. Tumor uptake rates of [^18^F]F-FGFR1 in RT-112 tumor-bearing mice at 30 min, 60 min and 120 min post-injection were 3.84 ± 0.17%ID/g, 2.60 ± 0.10%ID/g and 0.98 ± 0.06%ID/g, respectively, which were significantly higher than those in A549, SNU-16, Calu-3 and RT-112-blocked xenograft mice (*P* < 0.001).

**Table:** Table 1 Biodistribution of [18F]F-FGFR1 in RT-112 xenografts (n=5).

Organ (Mean ± SD, %ID/g)	30 min	60 min	120 min
**Brain**	0.66 ± 0.05	0.69 ± 0.03	0.03 ± 0.01
**Heart**	1.02 ± 0.11	0.75 ± 0.06	0.10 ± 0.03
**Lung**	0.75 ± 0.11	0.65 ± 0.09	0.14 ± 0.05
**Liver**	1.35 ± 0.23	1.43 ± 0.06	0.17 ± 0.08
**Kidney**	9.44 ± 0.58	6.85 ± 0.77	18.97 ± 4.82
**Muscle**	0.24 ± 0.05	0.28 ± 0.03	0.09 ± 0.02
**Bone**	0.33 ± 0.10	0.35 ± 0.12	0.48 ± 0.23
**Tumor**	3.84 ± 0.17	2.60 ± 0.10	1.35 ± 0.07

**Table:** Table 2 Biodistribution of [18F]F-FGFR1 in A549 xenografts (n=5).

Organ (Mean ± SD, %ID/g)	30 min	60 min	120 min
**Brain**	0.61 ± 0.06	0.72 ± 0.13	0.64 ± 0.17
**Heart**	0.90 ± 0.09	0.88 ± 0.04	0.64 ± 0.07
**Lung**	0.69 ± 0.12	0.65 ± 0.04	0.53 ± 0.07
**Liver**	1.53 ± 0.11	1.52 ± 0.18	1.58 ± 0.08
**Kidney**	9.35 ± 1.87	7.89 ± 1.06	4.46 ± 0.60
**Muscle**	0.27 ± 0.03	0.27 ± 0.02	0.28 ± 0.02
**Bone**	0.33 ± 0.10	0.35 ± 0.06	0.39 ± 0.02
**Tumor**	0.87 ± 0.08	0.64 ± 0.08	0.53 ± 0.04

**Table 3:** Biodistribution of [^18^F]F-FGFR1 in SNU-16 xenografts (n=5).

Organ (Mean ± SD, %ID/g)	30 min	60 min	120 min
**Brain**	0.56 ± 0.06	0.60 ± 0.07	0.56 ± 0.10
**Heart**	0.70 ± 0.09	0.62 ± 0.09	0.55 ± 0.06
**Lung**	0.67 ± 0.08	0.60 ± 0.03	0.46 ± 0.09
**Liver**	1.54 ± 0.11	1.57 ± 0.12	1.45 ± 0.10
**Kidney**	8.04 ± 0.97	7.43 ± 0.83	4.23 ± 0.70
**Muscle**	0.27 ± 0.06	0.27 ± 0.03	0.31 ± 0.02
**Bone**	0.38 ± 0.10	0.37 ± 0.05	0.41 ± 0.02
**Tumor**	0.66 ± 0.05	0.60 ± 0.06	0.45 ± 0.04

**Table 4:** Biodistribution of [^18^F]F-FGFR1 in Calu-3 xenografts (n=5).

Organ (Mean ± SD, %ID/g)	30 min	60 min	120 min
**Brain**	0.60 ± 0.04	0.66 ± 0.06	0.67 ± 0.18
**Heart**	0.79 ± 0.08	0.80 ± 0.07	0.79 ± 0.17
**Lung**	0.62 ± 0.08	0.54 ± 0.06	0.48 ± 0.04
**Liver**	1.40 ± 0.07	1.54 ± 0.12	1.33 ± 0.17
**Kidney**	8.47 ± 0.83	7.48 ± 0.60	3.02 ± 0.69
**Muscle**	0.28 ± 0.04	0.25 ± 0.03	0.30 ± 0.04
**Bone**	0.42 ± 0.05	0.36 ± 0.05	0.37 ± 0.04
**Tumor**	0.59 ± 0.08	0.58 ± 0.06	0.43 ± 0.03

**Table 5:** Biodistribution of [^18^F]F-FGFR1 in RT-112-blocked xenografts (n=5).

Organ (Mean ± SD, %ID/g)	30 min	60 min	120 min
**Brain**	0.61 ± 0.03	0.61 ± 0.06	0.71 ± 0.10
**Heart**	0.74 ± 0.08	0.70 ± 0.07	0.75 ± 0.14
**Lung**	0.60 ± 0.05	0.59 ± 0.04	0.51 ± 0.07
**Liver**	0.67 ± 0.16	0.51 ± 0.09	0.50 ± 0.09
**Kidney**	9.35 ± 0.82	7.31 ± 0.87	3.37 ± 0.54
**Muscle**	0.26 ± 0.03	0.29 ± 0.03	0.27 ± 0.02
**Bone**	0.31 ± 0.07	0.37 ± 0.04	0.35 ± 0.07
**Tumor**	0.40 ± 0.02	0.39 ± 0.03	0.40 ± 0.02

Based on the micro-PET/CT scan, tumor to muscle ratio (T/M), tumor to liver ratio (T/L) and tumor to kidney ratio (T/K) were quantified. At each time point, T/M and T/K in RT-112 (FGFR1-high expression) xenografts were higher than those in other cell lines (FGFR1-low expression) and RT-112-blocked xenografts (*P* < 0.001). At 30 min and 60 min post-injection of [^18^F]F-FGFR1, RT-112 xenografts showed higher T/L than others (*P* < 0.001). However, at 120 min after [^18^F]F-FGFR1 injection, the blocked group exhibited a higher T/L value than RT-112 xenografts (0.62 ± 0.09%ID/g *vs.* 0.85 ± 0.22%ID/g, *P* = 0.002), as well as significantly higher T/L value than other cell line xenografts (*P* < 0.001). These results showed that [^18^F]F-FGFR1 could visualize FGFR1 expression in xenografts with clear imaging, especially at 30 min post-injection.

## Discussion

In malignant tumors, as in all progressive diseases, early and reliable diagnosis is critical to prolong survival and improve prognosis. PET/CT, combining anatomical imaging with functional imaging, has the clear advantage of a one-stop diagnosis service with high sensitivity and excellent spatial resolution (28). Molecular imaging techniques have revolutionized the field of oncologic diagnostics and therapeutics (29). The FGF/FGFR signaling pathway has been proved to be implicated to tumor growth, metastasis and recurrence (30), due to which it has being emerging as a hotspot of therapeutic target in the field of oncology (31). With the development of FGFR inhibitors, one of the major challenges is to identify patients with specific FGFR aberrations beforehand. Here, we aimed to synthesize an FGFR1-targeted radiotracer, which could be applied in the clinic to dynamically monitor the expression of FGFR1 and screen for patients who may benefit from FGFR1 inhibitors.

Firstly, by a conventional solid-phase synthesis method, FGFR1-targeting peptide was successfully synthesized. The sequences of the peptide were optimized by the introduction of polyethylene glycol (PEG). Previous studies have reported the extensive applications of PEG in the modification of targeted peptide sequences. PEGylation was clarified to have the ability to improve the pharmacokinetic profiles of the peptides by reduce the uptake and the excretion by hepatobiliary system (32). Then the newly synthesized FGFR1-targeting peptide was labeled with fluorine-18, after which the peptide was tested to stay with good radiochemical purity and stability over time. These results indicated that the probe was not decomposed by enzymes during metabolism. A single elution peak was observed in the *in vivo* stability experiments. Comparing the retention time with that of fluorine-18, the identities of the major eluted peak were considered to be a metabolic fragment of the peptide. However, the nature and radioactivity of the fragments were unclear, and follow-up studies are needed to confirm this hypothesis.

Western blotting confirmed the expression of FGFR1-4 subtypes in different cell lines. Compared to the A549, SNU-16 and Calu-3 cell lines, the RT-112 cell line, which expresses high levels of FGFR1, showed rapid uptake, which could be blocked by excess unlabeled peptide. This finding indicated that [^18^F]F-FGFR1 can specifically bind to its corresponding receptor subtype (FGFR1). The *K*_D_ value of [^18^F]F-FGFR1 was 0.19 ± 0.03 µM. The *K*_D_ value in this study indicated a higher affinity and was close to the value reported by Hansen (0.17 ± 0.06 µM) (27). The labeling of the positron nuclide fluorine-18, chelators and PEG groups has also been demonstrated to not alter the chemical properties of the peptide. [^18^F]F-FGFR1 was suggested to have great potential to serve as a novel molecular probe to target FGFR1 for imaging. [^18^F]F-FGFR1 was found to have a lower internalization rate. The internalization of radiotracers is generally recognized to be affected by a variety of factors. In addition to our study, another previous research has also drawn the same conclusion that no significant connection between the uptake rate and internalization rate was found (33).

Based on the images and biodistribution obtained from micro-PET/CT imaging, RT-112 (FGFR1-high expression) xenografts displayed significantly higher tumor uptake of [^18^F]F-FGFR1, as well as higher T/M, T/L and T/K values than those in A549, SNU-16 and Calu-3 xenografts (FGFR1-low expression), especially at 30 min after injection. The radioactivity of [^18^F]F-FGFR1 mainly detected in the kidney and FGFR1-positive tumors. In normal organs, the novel radiotracer [^18^F]F-FGFR1 showed no or very low uptake. The high-contrast images elucidate new perspectives for detecting FGFR1-positive malignant foci in these organs, especially in the brain and liver. These results also illustrated that [^18^F]F-FGFR1 could be cleared rapidly in plasma, which supports early imaging. The optimal temporal window of [^18^F]F-FGFR1 PET/CT imaging is likely 30 min post-injection.

The specific FGFR1-targeting ability of [^18^F]F-FGFR1 has been verified in a receptor blocking experiment. Compared to RT-112 xenografts, the RT-112 blocked group showed not only a lower tumor uptake, T/M value and T/K value at each time point, but also lower T/L value at 30 min and 60 min post-injection. At 120 min post-injection, the T/L value in the RT-112 blocked group was higher than that in the unblocked group. The first possible reason was the clearance of the radiotracer. Referring to the micro-PET/CT imaging and biodistribution data, the tumor uptake of [^18^F]F-FGFR1 in RT-112 xenografts decreased over time. Moreover, the liver has long been recognized as a unique organ with a high regenerative capacity following injury. It has been associated with the action of different growth factors, not only epidermal growth factor receptor (EGFR) and hepatocyte growth factor (HGF) but also FGF/FGFR signaling (34). Theoretically, excess of unlabeled peptide saturated the receptor binding sites on the tumor cell surface, meanwhile it bond to the FGFR1 in liver. The decrease in radioactivity uptake in the liver caused a relatively high T/L in RT-112-blocked xenografts.

In our study, [^18^F]F-FGFR1 showed high stability, affinity, specificity and good imaging capacity in cells and tumor-bearing nude mice overexpressing FGFR1, which indicated promise for detecting FGFR1 expression by noninvasive methods. This study was subject to limitations that need to be addressed in the future in parallel. Firstly, the mechanisms of internalization and the subcellular distribution of the tracers remain unclear. The imaging temporal window is relatively short. Secondly, according to Hansen’s study, other FGFR1-targeting sequences may have better affinity or slower dissociation (27). Additional researches may be required to figure out whether there are correlations among better affinity, slower dissociation and better imaging effect.

## Conclusions

In this study, we successfully synthesized the FGFR1 peptide and then radiolabeled it with fluorine-18. The radiolabeled FGFR1 peptide not only presented high stability, affinity and specificity, but also showed clear imaging with high T/M contrast in tumors that expressed high levels of FGFR1. In spite of shortcomings, such as short imaging temporal window, low internalization rate, [^18^F]F-FGFR1 is a promising and potential novel molecular probe for visualizing FGFR1 expression *in vivo*, which possesses prominent significance in future clinical application.

## Data availability statement

The original contributions presented in the study are included in the article/supplementary material. Further inquiries can be directed to the corresponding author.

## Ethics statement

All animal experiments were carried out in accordance with the Laboratory Animal Ethical Review of Animal Guidelines of China and strictly adhered to the Principles of the Laboratory Animal Ethical Committee of the Fourth Hospital Hebei Medical University (No. 2020016).

## Author contributions

All authors contributed to the article and approved the submitted version. Material preparation, data collection and analysis were performed by YC, JH, YZ, XZ and MZ. Data curation was performed by JZ and JW. The first draft of the manuscript was written by YC. The manuscript writing-review and editing were done by XZ. All authors contributed to the article and approved the submitted version.
